# Wave Characteristics in Breaststroke Technique with and Without Snorkel Use

**DOI:** 10.2478/hukin-2013-0081

**Published:** 2013-12-31

**Authors:** Ana Conceição, António J. Silva, José Boaventura, Daniel A. Marinho, Hugo Louro

**Affiliations:** 1 Sport Sciences School of Rio Maior, Rio Maior, Portugal.; 2 University of Trás-os-Montes and Alto Douro, Vila Real, Portugal.; 3 Research Center in Sports Science, Health and Human Development, Vila Real, Portugal.; 4 INESC TEC - INESC Technology and Science (formerly INESC Porto) and ECT - School of Science and Technology, University of Trás-os-Montes e Alto Douro, Portugal.; 5 University of Beira-Interior, Covilhã, Portugal.

**Keywords:** competitive swimming, stroke rate, stroke length, swimming snorkel, wave motion, Fourier analysis

## Abstract

The purpose of this paper was to examine the characteristics of waves generated when swimming with and without the use of Aquatrainer® snorkels. Eight male swimmers performed two maximal bouts of 25 m breaststroke, first without the use of a snorkel (normal condition) and then using a snorkel (snorkel condition). The body landmarks, centre of the mass velocity, stroke rate, stroke length, stroke index, and Strouhal number (St) were quantified. Fourier analysis was conducted to determine the frequency, amplitude, and phase characteristics of the vertical undulations. We also determined the undulation period, the first and second harmonic wave percentage, and the contribution of these components to the power of each of the wave signals. The first wave harmonics had a frequency of 0.76 Hz (normal condition) and 0.78 Hz (snorkel condition), and the second wave harmonics had a frequency of 1.52 Hz (normal condition) and 1.56 Hz (snorkel condition). Under the normal conditions, the wave amplitude was higher on the vertex (0.72 m) and cervical (0.32 m) than that produced under snorkel conditions (0.71 m and 0.28 m, respectively). The lowest values were found in the hip (0.03 m in normal conditions, and 0.02 m in snorkel conditions) and in the trunk (0.06 m in normal conditions, and 0.04 m in snorkel conditions). It can be concluded that snorkel use seems to lead to slight changes in the biomechanical pattern in swimming velocity, as well as several stroke mechanical variables.

## Introduction

The wave motion is evident in elite breaststroke swimmers, whose movements have been observed to be similar to those of aquatic mammals. The propulsive movements of dolphins have been studied by Videler and Kamermans (1985), who suggested differences between the upward and downward movements of dolphins. Specifically, they stressed that the downward movement causes more thrust than the upward movement, which can be explained by an increase in drag during upward action.

Ungerechts et al. (1998) found that while the swimming velocity of dolphins increases with kick frequency, amplitude and kick frequency are independent. Moreover, Hochtsein and Blickhan (2010) recently suggested that although the human body is limited by the asymmetry of its movement, it possesses as high flexibility as a body of a fish, allowing high-level swimmers to mimic the locomotion strategies of fish. They also discovered that several of the behaviours of aquatic animals could be mimicked to enhance the performance of swimmers.

Over the years, researchers have sought to identify various technical indicators that can improve swimming performance. Accordingly, researchers have developed studies to produce relevant information that can help swimmers and coaches to apply practical decisions with regard to the technical model that is used. Movement has commonly been regarded as comprising of sinusoids of a particular frequency and phase characteristics of the swimming stroke, generating the hypothesis that movement is controlled by “oscillator-like mechanisms” (Kelso et al., 1980; 1981). Thus, it is possible that an objective measure of the differences between movement patterns could be achieved by quantifying the fundamental waveforms and its frequency harmonics.

The study of the cephalous - caudal wave was started by Sanders et al. (1995; 1998), who used Fourier analysis to quantify the amplitude and phase of the vertical undulations of butterfly stroke swimmers. Sanders et al. (1995) showed that the vertical displacement-time profiles of the body parts of skilled butterfly swimmers are characterised by low frequency waveforms. These phase relationships result in a caudal wave traveling along the body during the stroke cycle. Sanders et al. (1998) also reported that the percentage of power contained in the fundamental frequency of the vertex, head and shoulder vertical undulations causes the swimmer to modify his technique from a conventional (flat) style to wave action. In other studies, the Strouhal number has been shown to be an indicator of efficiency that relates beat frequency and amplitude to swimming velocity (Triantafyllou and Triantafyllou, 1995; Fish and Ror, 1999; Arrelano et al., 2002).

While there have been numerous recent reports about the breaststroke swimming technique, there has been a lack of detailed studies examining the wave motions in breaststroke with the use of equipment such as a snorkel. The Aquatrainer® snorkel (K4 b2, Rome, Italy) allows the measurement of ventilation and oxygen uptake during swimming, and several studies have shown that this equipment recorded the cardiorespiratory parameters of swimmers with validity and accuracy (Toussaint et al., 1987; Keskinen et al., 2003; Rodriguez et al., 2008). Therefore, it is relevant to analyse the effects of snorkel use and its contribution and suitability as a training device. Barbosa et al. (2010) reported that the Aquatrainer® snorkel affected the normal biomechanical pattern of the breaststroke stroke. However, only a few studies have focused on the effect of the use of the snorkel on the wave characteristics of breaststroke.

Hence, the aim of the present study was to determine the effect of using a snorkel on the frequency, amplitude and phase characteristics of breaststroke using Fourier analysis and the calculation of the Strouhal number.

The objectives of this study were as follows: to determine and verify the home advantage in soccer in the UEFA zone in the first decade of the 21st century and its evolution during this time. We evaluated the behaviour of home advantage taking into account the UEFA ranking and described home advantage in the most powerful leagues of Europe.

## Material and Methods

### Participants

Eight national-level, male Portuguese swimmers who are experts in the breaststroke technique volunteered to participate in this study. The subjects average (SD) age, body height, body mass, arm span and FINA points for 100 m breaststroke were 21.25 ± 6.73 years, 1.77 ± 0.03 m, 71.14 ± 12.39 kg, 1.84 ± 0.03 m and 483.125 ± 118.38 points, respectively. All subjects gave their written informed consent before data collection, which was approved by a local ethics committee and performed according to the declaration of Helsinki.

### Measures

#### Procedures

The measures were performed in a 50 m indoor swimming pool. All subjects performed a warm-up of 400 m at medium intensity. After the warm-up, the subjects were instructed to perform two bouts of 25 m breaststroke swims at maximum intensity, one bout in a “normal condition” and another in the “snorkel condition”, using the Aquatrainer designed by COSMED®. The sequence for the implementation of the two conditions was randomly defined. Full recovery was allowed.

The subjects were used to training with snorkel devices. However, the subjects only started to use the Aquatrainer snorkel two weeks before the experiments.

### Data collection

The swimmers were videotaped in the sagittal plane in one cycle with a pair of cameras that provided both underwater projection (Sony Mini-DV, 50 Hz) and projection above the water surface (Sony Mini-DV, 50 Hz). The cameras were placed stationary at 25 m from the headwall, in a lateral wall of the swimming pool, perpendicular to the line of motion and 10 m away from the swimmer (Barbosa et al., 2010). The study comprised the kinematical analysis of one stroke cycle, where the frames were scanned manually using the APAS System (Ariel Dynamics, USA) at a frequency of 50 Hz.

The digitised images incorporated the definition of the anthropometric spatial model from Zatsiorsky-Seluyanov, adapted by de Leva (1996), in which the body is divided into the eight following segments: 1 - head, 2 – trunk, 3 – arm, 4 - forearm, 5 - hand, 6 – thigh, 7 - leg, 8 - foot (Pavol et al., 2002; Lafond et al., 2004; Hirata and Duarte, 2007; Barbosa et al., 2010). This was done using data of the mass and relative locations of the centres of mass of the different segments, allowing the calculation of the location of the swimmer’s centre of mass (Colman et al., 1998). The stroke cycle was measured between the 18 and 22 m and identified at the end of the leg recovery when the legs were in flexion and the orientation of the toes had returned to the starting position.

A two-dimensional analysis was conducted by creating a single image of a dual projection, as described previously (Barbosa et al., 2005; Vilas-Boas et al., 1997). The independent digitalisation from both cameras was reconstructed with the help of a calibration volume (cube with 12 calibration points) and a 2D DLT algorithm (Abdel-Aziz and Karara, 1971; Wilson et al., 1997). For the analysis of the centre of mass kinematics, a filter with a cut-off frequency of 5 Hz was used, as suggested by Winter (1990).

Stroke mechanics were assessed by the stroke rate (SR = 1/P, Hz), the stroke length (SL, m) and the average swimming velocity of the centre of mass (m/s). The swimming efficiency was estimated by the stroke index (SI = velocity × stroke length), as suggested by Costill et al. (1985).

The Strouhal number is a dimensionless number that represents the ratio of unsteady and steady motion (Fish and Rohr, 1999). The Strouhal number (St) can be defined by the following equation (1):

(1) $$\mathrm{St}=\frac{A_{p-p}f}{U}$$

where: A p-p is the tail-beat peak-to-peak amplitude (the distance from the peak of the tail fluke upstroke to the peak of the tail fluke downstroke), *f* is the tail-beat frequency and U is the average body velocity. It can also be interpreted as a reduced frequency that provides the ratio between the motion caused by the tail oscillation and that due to the forward motion of the swimmer. In such cases, it is an estimate of the unsteadiness in the fluid-body interaction relative to the overall fluid structure. The practical use of this number is to adjust its variables, in this case with frequency and amplitude values modifications, to bring its value closer to the more efficient range, without decreasing the swimmer’s speed. As such, it is an indicator of the efficiency in the aforementioned energy transfer mechanism.

The amplitude of the harmonics was analysed by the peak-to-peak amplitude. Fourier analysis was used to quantify the amplitude and phase of the vertical undulations of butterfly swimmers (Sanders et al., 1995). The FFT function was applied to the acquired time series containing the whole number of periods to generate an accurate frequency response. As an example, Figure 2 shows the plots of one period of one acquired signal and the 2 major harmonics computed and their sums (H1+H2). As shown, in this case, the reconstructed signal (H1+H2) is very close to the acquired one, and this procedure has produced results similar to the other processed signals.

To estimate the frequency spectrum, H (f), of a given continuous signal in time domain, h (t), the Fourier transform defined in the equation 2 can be used (Brigham, 1974):

(2) $$\text{H}({\text{f}}^{\prime })=\int_{-\infty }^{+\infty }\text{h}(\text{t}){\text{e}}^{-\text{j}2\pi \text{ft}}\text{dt}$$

However, in our study, as a result of the sampling process, the measured signals were not in continuous time. Instead, they were measured and recorded periodically in time with an interval of time between the data records designated by the sampling period (T). In this case, the signal, h(t), was represented by the discrete signal, h(KT), with N samples resulting from sampling the continuous signal, h(t), with a sampling frequency, fs, resulting in a sampling period of T=1/fs. In this work, the sampling period used was T = 0.02 s (Sanders et al., 1995; 1998). To estimate the frequency spectrum of the discrete signals in the time domain, h (kT), the DFT - Discrete Fourier transform algorithm was used (Brigham, 1974) defined by the equation 3:

(3) $$\text{H}(\text{n})=\sum_{\text{k}=0}^{\text{N}-1}\text{h}(\text{kt}){\text{e}}^{-\frac{\text{j}2\pi \text{nk}}{\text{N}}}$$

where: the discrete Fourier transform H(n), with the range from 0 to N-1, is a discrete function to approximate H (f), where N is the number of signal samples and T is the sampling period. Note that H(0),…, H(f), corresponds to the harmonics amplitudes at f=0 Hz,…, f=fs/N. In this work, the DFT of the studied signals was computed to determine the main components in frequency, i.e., the relevant harmonics, of various signals collected from a number (n=8) of swimmers. The DFTs were calculated using the fft function of the programming language, Matlab (MathWorks). This function implements the algorithm FFT - Fast Fourier Transform, which is a faster way to determine the discrete Fourier transform given in the previous expression (Brigham, 1974; Nussbaumer, 1981; Elliot and Rao, 1982). The analysis described above was previously used in other swimming investigations (Sanders et al., 1995; 1998).

The spectral power density, Phd, of the signal h(KT) between harmonics was calculated as described in equation 4:

(4) $${\text{P}}_{\text{hd}}=\text{H}(\text{n}).^{*}\text{conj}(\text{H}(\text{n})),{\text{P}}_{\text{hd}}=\text{fft}(\text{h}).^{*}\text{conj}(\text{fft}(\text{h}))$$

where: H(n), which is a vector of complex numbers, representing the temporal DFT of the signal h(KT), conj is the conjugate of a complex number and the operator (.*) denotes the element-by-element product of the considered vectors. To obtain the amplitude of each harmonic, equation 5 was employed:

(5) $${\text{P}}_{\text{ha}}=\text{abs}(\text{fft}(\text{h}))*2/\text{N}$$

**Figure 1:**
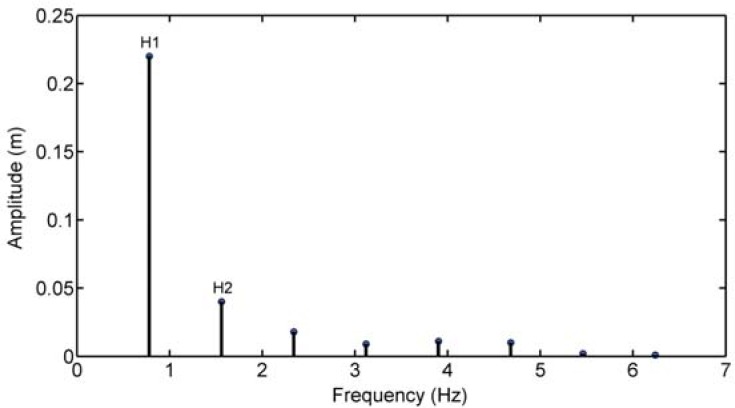
The amplitude signal (m) of the first to eighth harmonics of the vertex of subject 1 in the normal condition

**Figure 2:**
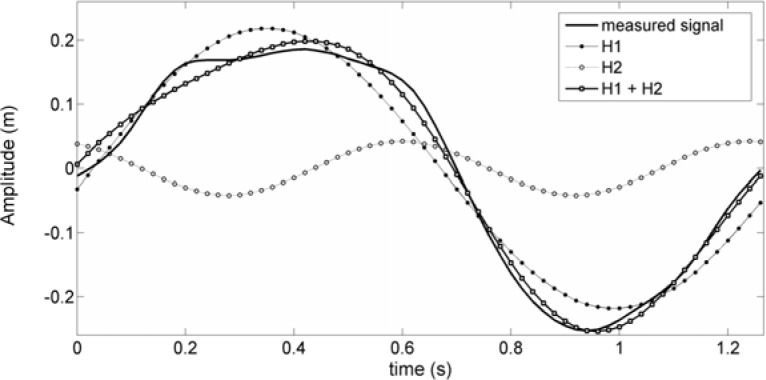
A plot of the measured signal (normal condition for the vertex of subject 1) and the computed harmonics (first and second harmonics). The signal sum of the first and second harmonics denotes the approximation to the measured signal when they are considered as the 2 more relevant harmonics

In this study, the power of the original signals was also determined and some of each of the relevant harmonics used to describe the original signals. In this case, the 2 major harmonics components are H1 and H2, as shown in the results section, Figures 1 and 2. The signal strength, Pot, provides a measure of its power and was determined by equation 6:

(6) $$\text{Pot}=\frac{1}{\text{N}}\sum_{0}^{\text{N}}{\text{y}}^{2}$$

We also calculated the percentages with which each body segment accounted for the power corresponding to the two most important harmonics presented in each of the original signals.

### Statistical Analysis

Statistical analyses were performed using SPSS 20.0 for Windows® (Chicago, IL). The variables were expressed as the means and standard deviations (SD). Normality and homoscedasticity assumptions were checked by Shapiro-Wilk and Levene tests, respectively. Data variation was analysed with the Wilcoxon Signed-Rank Test to assess differences between the two conditions (normal and snorkel). The Pearson correlation coefficient (r) was used to assess the association between variables to analyse the expected and unexpected associations between variables and to eliminate and / or confirm any unlawful associations between all the variables studied. The level of significance was set at p ≤ 0.05.

## Results

Table 1 presents the mean (SD) values of the assessed stroke mechanics in the 25 m breaststroke in both swimming conditions. The swimming velocity was significantly higher in the normal condition, 1.01(0.29) m/s, when compared to the snorkel condition, 0.91(0.30) m/s (p= 0.02). The stroke length had a tendency to decrease with the use of the snorkel and was 1.22(0.40) m/cycle, compared to 1.47(0.44) m/cycle in the normal condition. The stroke rate increased in the snorkel condition (0.76(0.11) Hz) when compared to the normal condition (0.71(0.15) Hz). The stroke index showed that the swimming efficiency was significantly higher in the normal condition (1.56(0.78) m2/c/s) than in the snorkel condition (1.19(0.74) m2/c/s), and (p=0.05).

Table 1 shows that the average frequency values of the first and second harmonic were similar in the two conditions and that the wave period in the normal condition in first harmonic varied slightly among swimmers. Furthermore, it was observed that the normal condition had higher values in the first and second harmonic compared with those in the snorkel condition. Additionally, the normal condition provided a slower wave formation compared to that in the snorkel condition. The wave period provided in SNK of the first harmonic varied slightly among swimmers.

Table 2 presents the average (SD) amplitude of the segments for all the swimmers in the normal condition and snorkel condition. The Fourier oscillation amplitudes were higher in the normal condition, except for the toe (0.14 m in the normal condition and 0.15 m in the snorkel condition). In the normal condition, the average amplitude values were higher on the vertex (0.72 m) and cervical (0.32 m) in comparison to those provided for the snorkel condition (vertex (0.71 m) and cervical (0.28 m)). In the two conditions, the lowest values were observed in the hip (0.03 m in the normal condition and 0.02 m in the snorkel condition) and trunk (0.06 m in the normal condition and 0.04 m in the snorkel condition).

By correlating the amplitude of the first harmonic in the normal condition and snorkel condition, we verified a correlation between the average of the amplitude for the first harmonic in the snorkel condition with that in the normal condition. This did not occur in the second harmonic (r=0.894; p= 0.03). Figure 1 illustrates the amplitudes of the harmonics 1 to 8 in the normal condition of the vertex signal for the SW1. The 2 relevant harmonics (first and second harmonics) were located near 0.78 Hz and 1.56 Hz, with maximal peak-to-peak amplitudes of 0.22 m and 0.04 m, respectively.

Figure 2 shows the plots of the measured signal and the first and second harmonic waves, as well as the sum of these two harmonics for the normal condition for the vertex of the SW1. As shown, the reconstructed signal, or the sum of first and second harmonics, is very close to the measured signal.

Considering that the signal power is 100 percent, it was found that the contribution of each of the two major harmonics, i.e., the first and second harmonics, to the signal power were 83% and 8.5%, respectively. This means that these two major harmonic components can describe approximately 92 percent of the original wave characteristics.

By correlating the centre of mass velocity with the Fourier waves amplitudes for all the body segments in the two fundamental frequencies (first and second harmonics) in the normal condition, significant values were only observed in the second harmonic. There was a negative correlation between the centre of mass velocity and the knee amplitude, which means that a lower knee amplitude corresponds to a greater velocity of the centre of mass. By correlating velocity with the amplitude of Fourier undulations for all the body segments in both fundamental frequencies (first harmonics) in the snorkel condition, we found that there was a correlation between the shoulder amplitude and the velocity. Additionally, in the second harmonic, there was a correlation with the knee extent.

From Table 3, it can be observed that the high percentage of the total wave power is contained in the Fourier Fundamental Frequency (first harmonics) of the shoulder, cervical, knee and trunk in the normal condition, and the cervical, shoulder and knee condition in the snorkel condition. This means that the vertical motions of the cervical and knee were simply particular phases of a sinusoid oscillation (Sanders et al., 1995; Sanders, 1995).

The fundamental frequency of Fourier (H2) had higher values in both conditions in the toe, rather than in the ankle, knee (normal condition), and hip (snorkel condition). The correlation of the Fourier frequency (H2) with the velocity showed a correlation between the velocity and the knee, with a significance level of 0.01. This suggests that the power percentage contained in the knee is a crucial factor affecting the swimming velocity.

With respect to the correlation between the percentages of power contained in each body segment, for the Fourier frequency (first harmonics) and the velocity provided by the snorkel condition, there was a correlation between the velocity and shoulder, with a significance level of 0.01. As such, it seems that the percentage of power contained in the shoulder significantly affected the velocity, while the Fourier frequency (H2) did not.

On average, the Strouhal number (St) was equal to 0.41 in the normal condition and 0.53 in the snorkel condition, with an average amplitude of 0.12 m in the normal condition and 0.08 to 0.18 m in the snorkel condition. By correlating the St in the normal condition and snorkel condition with the amplitude, kick frequency and velocity, we found a negative correlation between the velocity and St in the normal condition (r =− 0.783, p = 0.021) and in the snorkel condition (r =− 0.830, p = 0.011), indicating that the most efficient swimmer presents the lowest St and consequently, the greatest velocity.

## Discussion

The aim of the present study was to determine the effect of using a snorkel on the frequency, amplitude and phase characteristics of breaststroke swimming using Fourier analysis and the calculation of the Strouhal number. The main finding of this study was that the use of a snorkel affects breaststroke swimming efficiency, which may involve motions that are characterised by a small number of composite waves that may be transmitted in a cephalous-caudal direction along the body to conserve mechanical energy.

It should be noted that in stroke mechanics, an increase in the stroke rate will cause the normal biomechanical pattern between velocity, stroke rate, and stroke length to be higher, while stroke length will tend to decrease (Barbosa et al., 2010). In this study, the normal behaviour was altered in the snorkel condition, as seen by the decrease in velocity and the increase in stroke length and stroke rate compared to the normal swimming condition. There was also a change in velocity in favour of the normal condition, which was expected. However, in the normal condition, the stroke rate decreases as the velocity increases. The decreased velocity observed during the snorkel condition can be attributed to a higher active drag (Barbosa et al., 2010). This means that in snorkel swimming, swimmers must perform relatively more work in terms of the stroke rate to obtain lower velocity compared to normal swimming. These results are similar to that reported by Kjendlie et al. (2003). Nevertheless, there was a tendency for a change in those variables when all the subjects were swimming with the snorkel. High stroke index values are inversely associated with the energy cost of swimming (Costill et al., 1985). As such, the lower stroke index in snorkel swimming must be related to the lower velocity that swimmers could achieve when swimming in this condition. However, in the normal condition, swimmers achieved a slightly higher velocity and therefore a greater speed.

In both swimming conditions, the pattern of the vertical movement of the body segments of the swimmers was almost entirely comprised of two low-frequency waveforms throughout the body. This suggests that these swimmers performed the breaststroke in a harmonic or wave-like pattern, as suggested by Ungerechts (1982), Thornton (1984) and Sanders et al. (1995). In this study, the amplitude undulation of the hip, knee, ankle and toe was small compared to the vertex, cervical and shoulder, contrary to that suggested by Thornton (1984) and Sanders et al. (1995). In the snorkel condition, the correlation between the velocity and the amplitude (H2) with the amplitude of the knee seems to indicate that a higher amplitude in the knee during snorkel swimming will lead to a higher swimming velocity. The evidence of wave motion travelling around the body (cephalous-caudal wave) is very important as according to Sanders et al. (1995), the waves transmit energy. This supports the idea that energy accrued by raising the upper body was reused to aid propulsion or reduce drag. The results showed that a wave with a frequency equivalent to the first harmonic is higher in the snorkel condition than in the normal conditions. The same occurs for the second harmonic, which may be explained by the constrains of the equipment used by the swimmers (Aquatrainer® snorkel).

As observed from positive correlation between the total power contribution percentage with the velocity and the shoulder of snorkel swimming in first harmonic, the power contribution of the shoulder determines increases or maintenance of velocity, and contributes to the propulsion of the swimmer. The Strouhal number is an integral part of the fundamentals of fluid mechanics and a dimensionless number that describes the oscillation. Differences in St were observed in the two conditions, with higher values in the snorkel condition. The values found in this study (mean of St = 0.41 for normal condition, and St = 0.53 for snorkel condition) are far from those achieved by efficient animals, such as fish and dolphins (between St = 0.25 and St = 0.35), as presented by Triantafyllou and Triantafyllou (1995), but close to the result obtained with top swimmers (St=0.80) as presented by Arrelano et al. (2002; 2003).

The practical use of this number is adjusted to its contributing variables to bring its value closer to a more efficient range without decreasing the swimmer’s speed. In this case, by introducing modifications in frequency and amplitude values, we can observe that with the use of a snorkel, the movement of a swimmer is very different from the excellent efficiency achieved during the normal pattern of swimming. Normal condition values are more similar to the values obtained by fish and dolphins. The significant changes in the amplitudes of the vertical undulations, the frequency and the power contribution indicated that swimmers with well-established movement patterns will adopt a new movement patterns with the use of the snorkel. These changes in wave motion may impose considerable demands on the sensorimotor system of the swimmers (Sanders et al., 1995).

In conclusion, the use of the Aquatrainer® snorkel in the breaststroke leads to slight changes in the biomechanical pattern in swimming velocity and in several stroke mechanical variables. However, more data and a possible comparison with wave and conventional breaststroke will be required to confirm these findings. Therefore, some caution should be used when analysing data of breaststroke technique involving the use of a snorkel.

## Figures and Tables

**Table 1:** Summary of stroke mechanics, frequencies, and period of undulations (n=8)

	Normal condition	Snorkel condition	*p*
Velocity (m/s)	1.01(0.29)	0.91(0.30)	0.02
SL (m/cycle)	1.47(0.44)	1.22(0.40)	0.23
SR (Hz)	0.71(0.15)	0.76(0.11)	0.55
SI (m^2^c/s)	1.56(0.78)	1.19(0.74)	0.05
Frequency (Hz) First Harmonic	0.76(0.06)	0.78(0.07)	0.15
Frequency (Hz) Second Harmonic	1.52(0.11)	1.56(0.15)	0.18
Period (s) First Harmonic	1.33(0.11)	1.29(0.12)	0.15
Period (s) Second Harmonic	0.66(0.05)	0.65(0.06)	0.17

*Notes*: SL= stroke length, SR= stroke rate, SI= stroke index.

**Table 2:** Summary of the amplitude of the first and second harmonic for all the segments in both swimming conditions

Body segments	Normal condition	Snorkel condition
Vertex	0.72(0.24)	0.71(0.22)
Cervical	0.32(0.19)	0.28(0.15)
Shoulder	0.18(0.08)	0.17(0.07)
Trunk	0.06(0.03)	0.04(0.01)
Hip	0.03(0.01)	0.02(0.01)
Knee	0.10(0.02)	0.10(0.03)
Ankle	0.12(0.03)	0.12(0.02)
Toe	0.14(0.04)	0.15(0.04)

**Table 3:** Summary of the percentage power contributions of the total power wave signals of the body segments.

	First harmonic component		Second harmonic component	

Body segments	Normal condition	Snorkel condition	Normal condition	Snorkel condition
Vertex	51.58(0.42)	54.42(0.41)	9.97(0.10)	10.53(0.05)
Cervical	84.40(0.11)	87.74(0.05)	8.03(0.05)	8.07(0.04)
Shoulder	91.64(0.31)	87.30(0.11)	2.91(0.03)	3.20(0.03)
Trunk	82.70(0.14)	64.14(0.31)	5.65(0.07)	9.05(0.10)
Hip	81.03(0.29)	61.80(0.30)	9.21(0.07)	15.77(0.10)
Knee	85.49(0.29)	74.95(0.31)	11.64(0.06)	10.59(0.07)
Ankle	69.34(0.09)	61.46(0.26)	19.77(0.10)	16.31(0.13)
Toe	65.57(0.11)	59.87(0.28)	20.53(0.12)	17.47(0.14)

## References

[b1-jhk-39-185] Abdel-Aziz YI, Karara HM (1971). Direct linear transformation from comparator coordinates into object space coordinates in close-range photogrammetry. Symposium on Close-Range Photogrammetry.

[b2-jhk-39-185] Alexander RM, Jayes AS (1980). Fourier analysis of forces exerted in walking and running. J Biomech.

[b3-jhk-39-185] Barbosa TM, Keskinen KL, Fernandes RJ, Colaço P, Lima AB, Vilas-Boas JP (2005). Energy cost and intracyclic variation of the velocity of the centre of mass in butterfly stroke. Eur J Appl Physiol.

[b4-jhk-39-185] Costill D, Kovaleski J, Porter D, Fielding R, King D (1985). Energy expenditure during front crawl swimming: predicting success in middle-distance events. Int J Sports Med.

[b5-jhk-39-185] De Leva P (1996). Adjustments to Zatsiorsky-Seluyanov’s segment inertia parameters. J Biomech.

[b6-jhk-39-185] Elliot DF, Rao KR (1982). Fast Transforms: Algorithms, Analyses and Applications.

[b7-jhk-39-185] Fish FE, Rohr JJ (1999). Review of dolphin hydrodynamics and swimming performance.

[b8-jhk-39-185] Hirata RP, Duarte M (2007). Effect of relative knee position on internal mechanical loading during squatting. Braz J Physical Therapy.

[b9-jhk-39-185] Hochtsein S, Blickhan R, Kjendlie P-L, Stallman TK, Cabri J (2010). Human Undulatory Swimming: Kinematics, Flow and Mechanical Model. Biomechanics and Medicine in Swimming XI.

[b10-jhk-39-185] Kelso JA, Holt KG, Flatt AE (1980). The role of proprioception in the perception and control of human movement: toward a theoretical reassessment. Percept Psychophys.

[b11-jhk-39-185] Kelso JA, Holt KG, Rubin P, Kugler PN (1981). Patterns of human interlimb coordination emerge from the properties of non-linear, limit cycle oscillatory processes: theory and data. J Mot Behav.

[b12-jhk-39-185] Kjendlie PL, Stallman R, Stray-Gundersen J, Chatard JC (2003). Influences of breathing valve on swimming technique. Biomechanics and Medicine in Swimming IX.

[b13-jhk-39-185] Rodríguez FA, Keskinen KL, Kusch M, HoVmann U (2008). Validity of a swimming snorkel for metabolic testing. Int J Sports Med.

[b14-jhk-39-185] Sanders R, Cappaert JM, Devlin RK (1995). Wave characteristics of butterfly swimming. J Biomech.

[b15-jhk-39-185] Sanders R, Cappaert JM, Pease DL (1998). Wave characteristics of Olympic breaststroke Swimmers. J Appl Biomech.

[b16-jhk-39-185] Thornton KM (1984). Learning from the Olympians: Butterfly stroke rhythm. Swimming World.

[b17-jhk-39-185] Toussaint H, Meulemans A, De Groot G, Hollander AP, Schreurs A, Vervoon K (1987). Respiratory valve for oxygen uptake measurement during swimming. Eur J Appl Physiol.

[b18-jhk-39-185] Triantafyllou GS, Triantafyllou MS (1995). An efficient swimming machine. Scientific American.

[b19-jhk-39-185] Ungerechts BE, Hollander AP (1982). A comparison of the movements of the rear parts of dolphins and butterfly swimmers. Biomechanics and Medicine in Swimming.

[b20-jhk-39-185] Ungerechts BE, Daly D, Zhu JP (1998). What dolphins tell us about hydrodynamics. J Swim Res.

[b21-jhk-39-185] Videler J, Kamermans P (1985). Differences between upstroke and downstroke in swimming dolphins. J Exp Biol.

[b22-jhk-39-185] Vilas-Boas JP, Cunha P, Figueiras T, Ferreira M, Duarte J, Daniel K, Hoffman U, Klauck J (1997). Movement analysis in simultaneously swimming technique. ölner Schwimmsporttage.

[b23-jhk-39-185] Wilson DJ, Smith BK, Gibson JK (1997). Accuracy of reconstructed angular estimates obtained with the Ariel Performance Analysis System. American Physical Therapy Association.

